# An Interdisciplinary Treatment Approach for Congenitally Missing Teeth: A Case Report

**DOI:** 10.7759/cureus.70106

**Published:** 2024-09-24

**Authors:** Rahul Ravi, Kushal P Taori, Vikrant V Jadhav, Mohammad Shafae Azmi

**Affiliations:** 1 Orthodontics and Dentofacial Orthopaedics, Sharad Pawar Dental College, Wardha, IND; 2 Oral Pathology and Microbiology, Sharad Pawar Dental College, Wardha, IND

**Keywords:** congenitally missing teeth, fixed dental prosthesis, hypodontia, orthodontic management, treatment choices

## Abstract

The absence of teeth from birth, also known as congenitally missing teeth (CMT) or hypodontia, is quite common, and generally, treatment is expensive. Patients with missing teeth can have more than just a poor appearance. They might also experience malaligned teeth, gum damage, poor jawbone growth, trouble chewing, unclear speech, and other issues. Treatment is often expensive and involves multiple specialties. This common but costly issue is important to many dental specialties, including orthodontics, pediatric dentistry, prosthodontics, periodontics, maxillofacial surgery, anatomy, and even health insurance companies. The causes, skeletal changes, frequency, risk factors, occurrence patterns, and treatment options for CMT are explained in this case report. This indicates that CMT usually shows up in permanent dentition and in females. Whether it tends to occur more frequently in the mandible or maxilla, as well as in the anterior vs posterior portions, is a matter of debate. It can come with several issues; thus, professional teams should respond to it appropriately. The following study presents the interdisciplinary treatment plan of a 28-year-old female girl presenting with a missing mandibular left central incisor and right mandibular lateral incisor. The treatment plan is interdisciplinary and includes orthodontic treatment and prosthetic rehabilitation.

## Introduction

The congenital absence of one or a few teeth is defined as “hypodontia” [1]. Oligodontia and anodontia are more serious types of dental agenesis (means lack or failure of development). Oligodontia involves missing more than six teeth, while anodontia means no teeth are present at all. These conditions are often linked to systemic disorders like Down syndrome, ectodermal dysplasias, and Ellis-van Creveld syndrome [2]. There are many theories about why dental agenesis happens. It could be because of how things change over time, or it might be due to things in the environment or body, like injuries, swelling, infections in the jaw, or hormonal imbalance [3]. Heredity genetics or family patterns can be the main reason. Besides the absence of lower incisors, issues with the formation of the anterior region of the mandible can also affect tooth growth [3]. It’s not common to have missing lateral and central incisors in the lower jaw; globally, about 6.1% of people are missing central incisors, and 4.3% are missing lateral incisors, excluding the third molars [4]. As per Polder et al., the teeth most commonly absent or missing are the lower second premolars, followed by the upper lateral incisors and upper second premolars [5]. They also mention that the occurrence of absent teeth can vary from 3% to 6.3%, and it tends to be higher in females than males [5,6]. Several earlier study works have shown the congenital absence of permanent lower front teeth and also a change in the actual morphology of mandibular symphysis [7]. Some studies have also shown that congenital missing incisors are accompanied by a smaller lower jaw or spacing in teeth, and there is retroclination of mandibular alveolar bone. These findings help to conclude that treatment needs orthodontic consideration in the steps of treatment planning [8]. Newman and Newman outlined three approaches for treating patients with missing lower incisors: forward moving the lower canines and posterior teeth to close the space, removing upper premolars to match the absent lower incisors, or creating space for an FPD or prosthetic teeth/restoration supported by implant [9]. The decision between space opening and closure is based on several factors, including the patient’s age, the shape and profile of their face, and their occlusal relationship. The aim of this study is to develop and implement an interdisciplinary treatment plan for a 28-year-old female patient presenting with a missing mandibular left central incisor and right mandibular lateral incisor. The treatment plan will focus on restoring function, aesthetics, and oral health through a coordinated approach involving multiple dental specialties. This study will also evaluate the outcomes of the chosen interventions and provide insights into the effectiveness of interdisciplinary collaboration in complex dental cases.

## Case presentation

**Figure 1:**
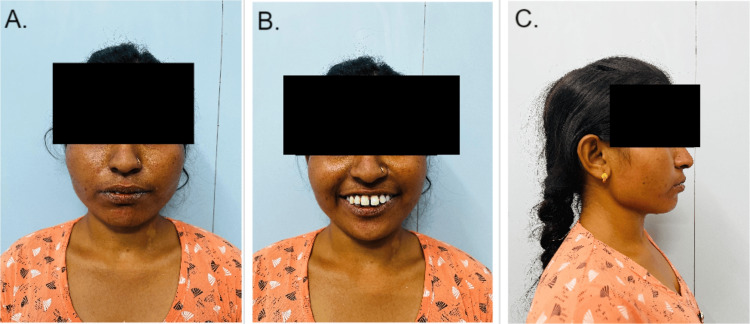
Extra-oral photos before treatment (A) The front profile of the patient with a closed lip. (B) The front profile of the patient with open lips and a smile, showing the proclination of maxillary anterior teeth. (C) Side profile of the patient.

**Figure 2:**
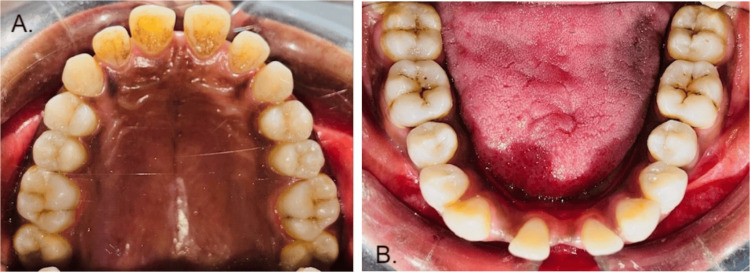
Intra-oral photos before treatment (A) Spacing in maxillary teeth before treatment. (B) Spacing in mandibular teeth before treatment.

**Figure 3:**
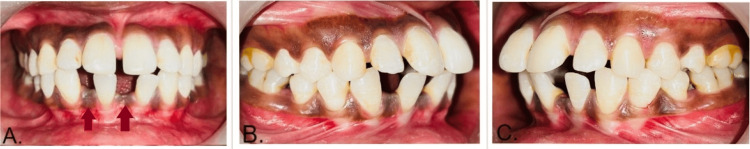
Intra-oral photos showing missing teeth, spacing, and proclination (A) Congenitally missing 31 and 42 denoted with red arrows. (B) Proclination of maxillary teeth and molar relation on the right side. (C) Proclination of maxillary teeth and molar relation on the left side.

The patient was a 28-year-old female who came to the outpatient department of Orthodontics and Dentofacial Orthopedics at Sharad Pawar Dental College and Hospital, Wardha, India. The patient presented with chief complaints of poor aesthetics, missing teeth in the lower front region, wide spacing in the lower front region, proclined front teeth, and incompetent lips. There was no significant medical, dental, or family history. The extraoral examination revealed a mesocephalic head form, mesoprosopic facial form with potentially incompetent lips, acute nasolabial angle, deep mentolabial sulcus, and convex profile, as shown in Figure 1. Intra-oral examination revealed midline diastema due to high labial frenal attachment and generalized upper and lower anterior spacing congenitally missing 31 and 42. Rotations present with 25, 34, and 44. Proclined upper and lower anterior, class I molar, and canine relation are shown in Figures 2, 3. After clinical extra-oral and intra-oral examination with radiographic evaluation, the case came to the diagnosis of Angle’s class I malocclusion with Dewey’s modification type 2 with congenitally missing left mandibular central incisor and right mandibular lateral incisor.

Treatment plan, progress, and treatment outcome

**Figure 4:**
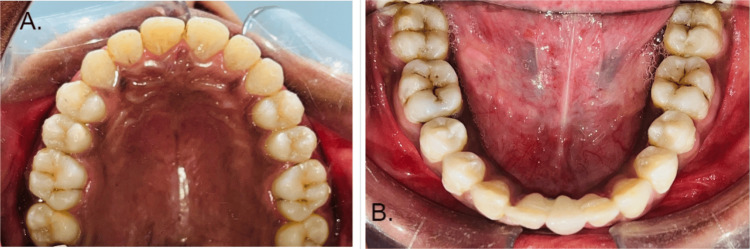
Intra-oral photos after treatment (A) A correction of spacing in maxillary teeth post-treatment. (B) A correction of spacing in mandibular teeth post-treatment.

**Figure 5:**
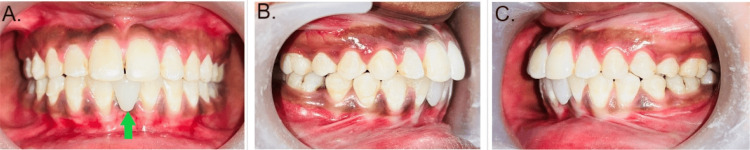
Intra-oral photos after treatment (A) The replacement of missing 31 with a prosthetic tooth denoted by a green arrow. (B) The correction of proclination in maxillary teeth and class I molar relation on the right side. (C) The correction of proclination in maxillary teeth and class I molar relation on the left side.

The first step of treatment was pre-treatment records. After that, initial leveling and alignment were done. Further correction of rotations was made, followed by space closure. The last step was the prosthetic rehabilitation of missing teeth, i.e., 31. Initial leveling and alignment were carried out with the help of initial round NiTi wires of 0.014 inches in diameter, followed by 0.016 inches in diameter round NiTi wire, which was further followed by rectangular NiTi wire of 0.019 × 0.025 inches. Post-leveling and alignment retraction were carried out by sliding mechanics on rectangular 0.19 × 0.025-inch SS wire. After the correction of proclination, the patient was referred to the Department of Prosthodontics for prosthetic rehabilitation of the missing 31. A Maryland bridge with 31 was given the closure of the midline diastema with correction of proclination in the upper and lower teeth. The class I molar and canine relation was achieved. Space closure was done in the lower arch, and the remaining space of missing 31 was replaced by the Maryland bridge. The normal overjet and overbite achieved are shown in Figures 4, 5.

## Discussion

An anomaly refers to something irregular, in contrast with what is considered to be normal. Disruptions in the epithelium and mesenchyme can significantly affect normal tooth development, resulting in dental anomalies. Depending on the developmental stage at which the disturbance occurs, different types of anomalies can arise, such as those affecting the number, structure, size, and/or shape of teeth [10]. Most dental anomalies develop during childhood. These developmental anomalies are classified based on abnormalities in their structure, eruption, location, texture, exfoliation, shape, and number [11]. Both local and systemic factors can contribute to these anomalies, which can originate before or after birth, potentially affecting both sets of teeth [12].

Congenital absence of the permanent right mandibular lateral incisor and left mandibular central incisor is not very rare. However, the main challenge is selecting the best treatment plan from the many available options. Definitive treatment can be performed as all the permanent dentitions have already erupted, and there are no retained deciduous teeth. The presence of major malocclusion symptoms, such as midline diastema, generalized spacing, rotation of nearby teeth, altered skeletal relation, and reduced chewing efficiency, can create a treatment dilemma. The choices include either creating space for prosthetic rehabilitation of the missing teeth or closing the space and using canines to replace the lower lateral incisors, avoiding further prosthetic treatment [13,14]. However, an interdisciplinary approach to achieving occlusion equilibrium and satisfactory functional aesthetics optimizes the outcome of comprehensive oral rehabilitation. This depends on various factors such as the number and position of absent teeth, the condition of the deciduous predecessors, overall alignment and occlusion, overjet and overbite, post-treatment maintenance, and, most importantly, the patient’s choice [15,16]. Prosthetic treatment includes an FPD, implant-supported restoration, resin-bonded bridges, and veneers. All preferences are considered before making an ortho-prostho interdisciplinary management. The space closure between the lower lateral incisor and canine is done with orthodontic treatment, and the missing lower central incisor is replaced prosthetically with a Maryland bridge, i.e., a resin-bonded bridge, keeping in mind the treatment cost and aesthetics. After comprehensive ortho-prostho management, achieving a balanced smile was satisfactory, especially since the patient’s main concern was having an unpleasant appearance. Apart from all this, facial height, facial profile, and gingival balance are created. Post-treatment periodontal health is also reviewed in follow-ups, and instructions for maintenance are given.

## Conclusions

Orthodontic management of any case with spacing is critical and interdisciplinary. Proper diagnosis and relevant treatment plan are paramount in treating the case with spacing and congenitally missing dentition. The case with spacing due to proclination in upper and lower dentition generally requires tipping movement of teeth to make them upright, which requires light continuous force.
